# Carbon nanosol-induced assemblage of a plant-beneficial microbiome consortium

**DOI:** 10.1186/s12951-023-02213-6

**Published:** 2023-11-20

**Authors:** Lingtong Cheng, Jiemeng Tao, Zechao Qu, Peng Lu, Taibo Liang, Lijun Meng, Wei Zhang, Nan Liu, Jianfeng Zhang, Peijian Cao, Jingjing Jin

**Affiliations:** 1 Beijing Life Science Academy, Beijing, 102200 China; 2 China Tobacco Gene Research Center, Zhengzhou Tobacco Research Institute of CNTC, Zhengzhou, 450001 China; 3 Key Laboratory of Ecological Environment and Tobacco Quality, Zhengzhou Tobacco Research Institute of CNTC, Zhengzhou, 450001 China; 4 China National Tobacco Quality Supervision and Test Center, Zhengzhou, 450003 China; 5 School of Agricultural Sciences, Zhengzhou University, Zhengzhou, 450001 China

**Keywords:** Carbon nanosol, Nano biofertilizer, Sustainable agriculture, PGPR, Plant growth, Microbiome

## Abstract

Carbon nanosol (CNS) is a carbon-based nanomaterial that promotes plant growth; however, its functional mechanisms and effects on the microbiome are not fully understood. Here, we explored the effects of CNS on the relationship between the soil, endophytic microbiomes and plant productivity. CNS treatment increased the fresh biomass of tobacco (*Nicotiana tabacum L.*) plants by 27.4% ± 9.9%. Amplicon sequencing analysis showed that the CNS treatment significantly affected the composition and diversity of the microbial communities in multiple ecological niches associated with tobacco, especially the bulk soil and stem endophytic microbiome. Furthermore, the application of CNS resulted in enhanced network connectivity and stability of the microbial communities in different niches, particularly in the soil, implying a strengthening of certain microbial interactions. Certain potentially growth-promoting root endophytic bacteria were more abundant under the CNS treatment. In addition, CNS increased the abundance of some endophytic microbial functional genes known to enhance plant growth, such as those associated with nutrient metabolism and the plant hormone biosynthesis pathways. We isolated two bacterial strains (*Sphingopyxis* sp*.* and *Novosphingobium* sp*.*) that were enriched under CNS treatment, and they were confirmed to promote tobacco plant growth in vitro. These results suggested that CNS might, at least in part, promote plant growth by enriching beneficial bacteria in the microbiome.

**Graphical Abstract:**

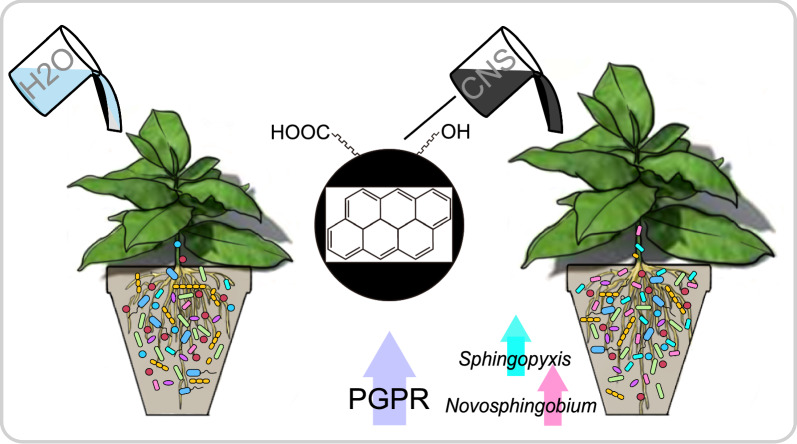

**Supplementary Information:**

The online version contains supplementary material available at 10.1186/s12951-023-02213-6.

## Introduction

With the rapid development of nanotechnology, various carbon nanomaterials have been widely studied and applied in agriculture and the life sciences. Some carbon-based nanomaterials have been found to promote plant growth, providing a promising new avenue for the development of sustainable agricultural practices [1]. Carbon-based nanomaterials can promote seed germination [2], root growth [3], and photosynthesis [4], whereas C60 and carbon nanotubes increase plant water retention, biomass, and fruit yield [5]. Carbon nanotubes have been used to promote root growth in onions (*Allium cepa*) and cucumbers (*Cucumis sativus*) [3], and graphene oxide has been used to enhance the content of auxin in tomato (*Solanum lycopersicum*) roots to promote plant growth [6]. Multiwalled carbon nanotubes affect tomato cell division and the expression of genes necessary for plant development [7, 8]. In addition, carbon nanoparticles can promote nutrient uptake and accumulation in mung beans (*Vigna radiata*), thus improving fertilizer efficiency and plant biomass [9]. Carbon nanotubes can increase soil urease and sucrase activity, soil organic carbon, and the available potassium content, thereby promoting plant growth [10]. Thus, nanomaterials have the potential to directly affect many physiological processes in plants.

Nanomaterials can also indirectly regulate plant growth or physiological characteristics by affecting the soil microbiome. The plant-associated microbiome, a so-called extended plant phenotype [11], plays important roles in nutrient acquisition, hormone production, and disease defense [12, 13]. These microorganisms regulate soil organic matter decomposition and soil mineral nutrient cycling and maintain soil fertility, which can directly or indirectly affect plant growth and mineral nutrient acquisition [14]. The possible mechanisms by which the plant microbiome influences plant growth can be divided into the following categories: (i) increasing nutrient utilization efficiency through phosphorus solubilization, nitrogen fixation, and iron carrier production; (ii) secreting plant hormones; (iii) producing volatile organic compounds (VOCs); (iv) inhibiting pathogens; and (v) stimulating the production of induced systemic resistance or antibacterial substances [15, 16].

Several studies have examined the effects of carbon-based nanomaterials on the plant rhizosphere microbiome, with possible consequences for crop performance. Exposure to carbon nanotubes reduces the amount of microbial carbon in the soil and decreases bacterial diversity [17], whereas high concentrations of carbon nanotubes reduce the activity of extracellular enzymes, which has a significant inhibitory effect on soil microorganisms. Nanocarbon black particles can affect the diversity of the soil microbiome, promoting nutrient uptake and plant growth in rye (*Secale cereale*) and sugar beet (*Beta vulgaris subsp. vulgaris*) [18].

Carbon nanosol (CNS) is a novel carbon-based nanomaterial that has shown great potential in regulating crop nutrient absorption, promoting plant growth, and increasing biomass accumulation [19]; however, the regulatory effects and growth-promoting mechanisms of CNS on microbial communities have not yet been elucidated. The aim of the present study was to decipher the relationships between the effects of carbon nanomaterials on plant-associated microbial communities and the resulting plant phenotype. We investigated the effects of CNS on tobacco growth characteristics and soil and endophytic microbial communities using amplicon sequencing (16S and ITS rRNA marker genes). Furthermore, two bacterial species that were enriched under CNS treatment were isolated, and their growth-promoting effects on tobacco plants were confirmed experimentally. Overall, this study provides valuable information for investigating the potential environmental applications of carbon nanomaterials as plant growth regulators.

## Results

### CNS promotes tobacco growth

**Fig. 1:**
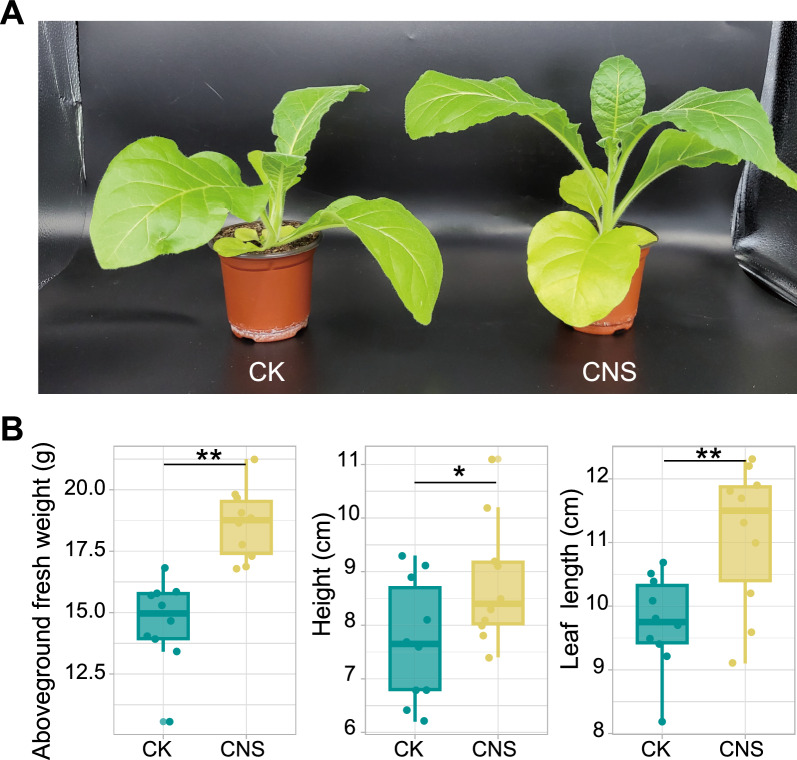
Effects of CNS on the growth of tobacco in a pot experiment. **A** Morphology of tobacco after 15 days of applying CNS at concentrations of 15 µg/mL three times to the soil in a pot experiment. **B** Effects of CNS on tobacco growth indicators. Asterisks denote significant differences (t test, *P* ≤ 0.05) between the CNS-treated and control (CK) samples treated with water, and two asterisks indicate *P* < 0.01

To further evaluate the role of CNS in enhancing plant growth, we investigated its effect on the growth of tobacco plants in soil (Fig. 1). As shown in Fig. 1A, tobacco growth was significantly enhanced by CNS in the pot experiment. Compared with the control, CNS increased the fresh weight by 27.4% ± 9.9%, height by 14.7% ± 14.2%, and leaf width by 13.9% ± 11.5% (Fig. 1B) after 15 days of CNS treatment; however, no difference was observed in the number of leaves between the CNS-treated and control samples. These results indicated that applying CNS to the soil through watering could effectively promote plant vegetative growth, thereby increasing the biomass of tobacco.

### CNS enhances the diversity of the bacterial and fungal microbiomes

**Fig. 2:**
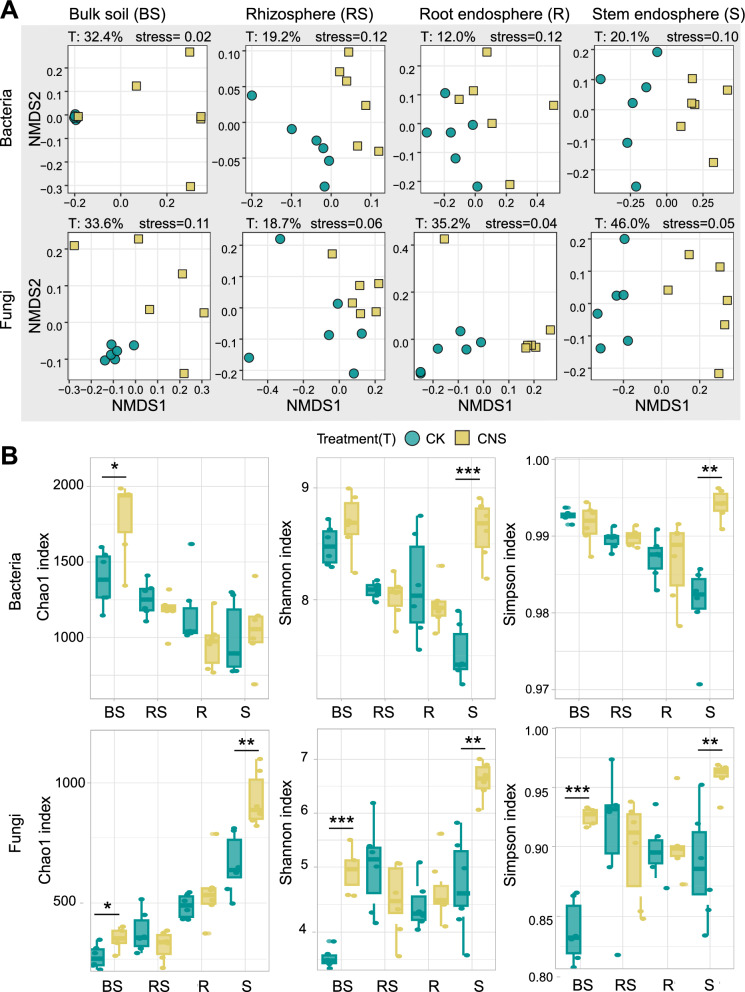
Diversity of tobacco-associated microbiomes. **A** Nonmetric multidimensional scaling (NMDS) ordinations based on weighted UniFrac distance matrices depicting the distribution patterns of bacterial and fungal communities in each compartment niche (for each niche, *n* = 12). The relative contribution of different factors to community dissimilarity was tested with PERMANOVA. “T” represents the effect of the CNS treatment. **B** Alpha-diversity of bacterial and fungal communities in soils (rhizosphere and bulk soils) and plant compartments (root endosphere and stem endosphere). Asterisks (“*”, *P* < 0.05; “**”, *P* < 0.01; “***”, *P* < 0.001) above the boxes indicate a significant difference between control and treatment within a compartment, determined using a nonparametric Kruskal–Wallis test

To evaluate the effect of CNS on the bacterial and fungal communities in the tobacco microbiome, we examined the changes in the microbial community in the soil (bulk soil and rhizosphere) and plant compartments (stem and root endosphere) two weeks after the application of CNS. A total of 18,117 bacterial amplicon sequence variants (ASVs; average of 114,684 reads per sample) and 10,012 fungal ASVs (average of 124,157 reads per sample) were identified from the 48 samples using amplicon sequencing (Additional file 2: Table S1). A nonmetric multidimensional scaling (NMDS) analysis based on Bray–Curtis distance showed that microbial communities in soil and plant compartments formed two distinct clusters regardless of CNS treatment (Additional file 1: Fig. S1). Our permutation multivariate analysis of variance (PERMANOVA) analysis indicated that the microbial niche explained the largest source of variation in both the bacterial and fungal communities (51.7% and 44.3%, respectively; *P* < 0.001), whereas the effect of CNS treatment on the microbial community was relatively small (2.6% and 3.5%, respectively; *P* < 0.001) (Additional file 1: Fig. S1). In contrast, the CNS treatment had a significant effect on both the bacterial and fungal communities (16–46%) in a specific ecological niche, with the greatest effect on the bulk soil microbial community (32.4% for bacteria and 33.6% for fungi) (Fig. 2A). The Chao1 richness of bacteria was higher in the bulk soil and lower in the stem endosphere, whereas the opposite trend was observed for the fungal community (Fig. 2B). The alpha-diversity of bacterial and fungal communities in the rhizosphere soil and root endosphere was not affected by CNS, whereas the bacterial and fungal diversity indices of the bulk soil and stem endosphere were significantly increased (*P* < 0.01) (Fig. 2B). In general, CNS led to shifts in the diversity and structure of the microbial community.

### Microbial community composition is influenced by CNS

**Fig. 3:**
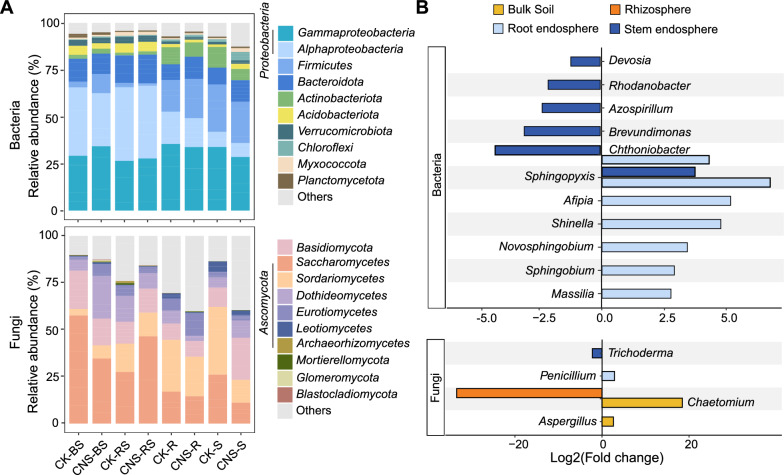
Community composition of soil- and plant-associated microbiomes. **A** Relative abundance of bacterial and fungal communities at the phylum level in the CNS-treated and control (CK) samples for four compartments: bulk soils (BS), rhizosphere (RS), root endosphere (R), and stem endosphere (S). **B** Fold change of bacterial and fungal genera related to plant growth–promoting ecological functions that were significantly affected by CNS

To better understand how CNS influences the microbial community, the relative abundances of bacteria and fungi at the phylum or class level were analyzed (Fig. 3A). The phylum *Proteobacteria* dominated the tobacco bacterial community, accounting for 36.74 ~ 66.46% of all ASVs, followed by *Firmicutes* and *Bacteroidota*. The abundance of *Alphaproteobacteria* in the CNS treatment was lower than that in the control (Additional file 2: Table S2). The tobacco soil and endophytic fungi were mainly distributed in five phyla, with Ascomycota being the most dominant (31.71–75.92%), followed by *Basidiomycota* (8.41–14.81%), *Mortierellomycota*, *Glomeromycota*, and *Blastocladiomycota*. After CNS treatment, the relative abundance of the *Saccharomycetes* and *Sordariomycetes* classes in the plant endophytic niche decreased.

Further analysis at the genus level showed that 29 and 40 bacterial genera were highly enriched in the tobacco root endosphere and stem endosphere, respectively, after CNS treatment, with 29 genera becoming less abundant in the stem endosphere (Additional file 1: Fig. S2). According to the relative abundances, the genera could be further divided into dominant (> 1%), common (0.1–1%), and rare (< 0.1%) genera. Most of those affected by the CNS treatment were dominant and common genera; the CNS treatment did not have a significant effect on most rare genera, affecting only 10 of them (Additional file 2: Table S3). Notably, CNS significantly elevated the abundance of genera related to plant growth, nitrogen fixation, and suppression of soil-borne diseases, such as *Sphingopyxis*, *Shinella*, *Massilia*, *Sphingobium*, and *Novosphingobium*, in the root endosphere (Fig. 3B). In addition, some genera related to organic compound degradation, such as *Chthoniobacter* and *Afipia*, and those related to crude oil and aromatic compound degradation, such as *Sphingopyxis* and *Novosphingobium*, were also enriched in the root endosphere after CNS treatment (Fig. 3B). In contrast, some bacteria were depleted after CNS treatment, such as *Brevundimonas*, *Azospirillum*, *Rhodanobacter*, and *Devosia,* in the stem endosphere. Some plant growth–promoting fungi [20–22], such as *Penicillium*, *Chaetomium*, and *Aspergillus*, were also enriched after CNS treatment, whereas *Trichoderma* showed a slight decrease in the stem endosphere (Fig. 3B). These results implied that CNS could restructure the microbial community, leading to a selective enrichment of beneficial microbes with the capacity to promote plant growth.

LDA effect size (LefSe) analysis was conducted at the genus level to determine the enrichment/depletion pattern between CNS-treated and control samples with an LDA score threshold of 2. The results revealed noticeable changes in fungal and bacterial abundance following CNS treatment (Additional file 1: Fig. S3). Specifically, three fungal genera, *Cladosporium*, *Talaromyces*, and *Alternaria*, were enriched after CNS treatment. Among these, *Cladosporium* showed enrichment in both tobacco bulk soil and stem endosphere after CNS treatment. On the other hand, *Candida* and *Trichoderma* decreased both in the stem and root endosphere after CNS treatment (Additional file 1: Fig. S3A). Additionally, *Burkholderia* was enriched in the bulk soil after CNS treatment (Additional file 1: Fig. S3B).

### Microbial ecological networks are affected by CNS

To evaluate the influence of CNS on potential interactions within the microbial community, we conducted a co-occurrence network analysis between the bacteria and fungi. The microbial interkingdom network pattern in the CNS-treated sample was similar to that in the control sample (Additional file 1: Fig. S4A); however, the proportion of negative edges in the network decreased slightly, from 24.4% to 21.7%, after CNS treatment (Additional file 1: Fig. S4A). Specifically, the bacterial community had higher network connectivity (i.e., network degree) than the fungal community, and there was no significant difference in network degree between the CNS treatment and control samples for the fungi or bacteria (Additional file 1: Fig. S4B). We further defined nodes with a high degree (> 50) and high closeness centrality (> 0.7) in the network as “hub nodes”. Compared with the control group (seven hub nodes), more hub nodes were identified after CNS treatment (27 hub nodes) (Additional file 2: Table S4), all of which were bacteria (Additional file 1: Fig. S4C). In other words, although the complexity of the interkingdom network was similar between the CNS treatment and control samples, the effects of some hub genera were strengthened under CNS treatment.

**Fig. 4:**
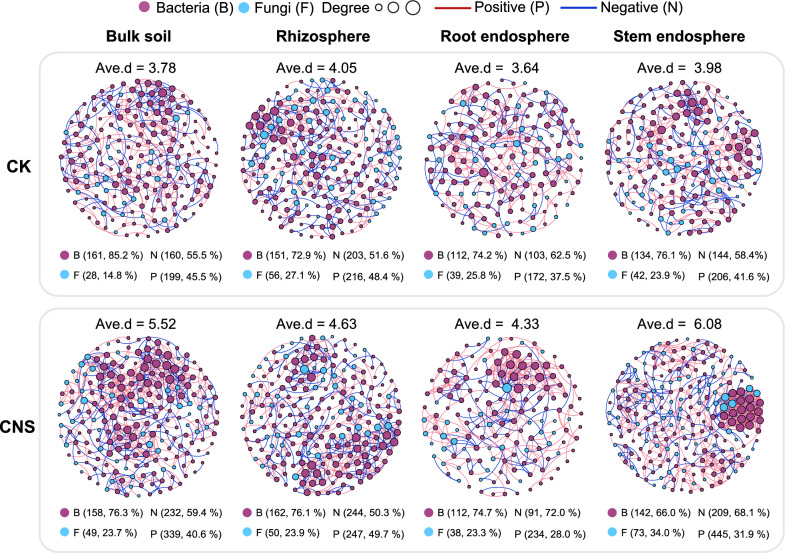
Microbial interkingdom networks within each niche. Co-occurrence network analysis showing microbial interkingdom network patterns differed clearly for CNS-treated and control (CK) samples in each plant niche; ave.d means the average degree of all nodes

In terms of niche, the complexity of the microbial networks was higher in the soil than in the endosphere, which was consistent with the diversity of the niches. The network connectivity of the microbial communities in all four niches was elevated after CNS treatment (Fig. 4). In addition, the increase in network topology indicators, including node number, edge number, and proportion of negative edges, indicated that CNS improved the complexity and stability of the tobacco microbiome ecological network (Fig. 4).

### CNS upregulates microbial genes with plant growth–promoting functions

**Fig. 5:**
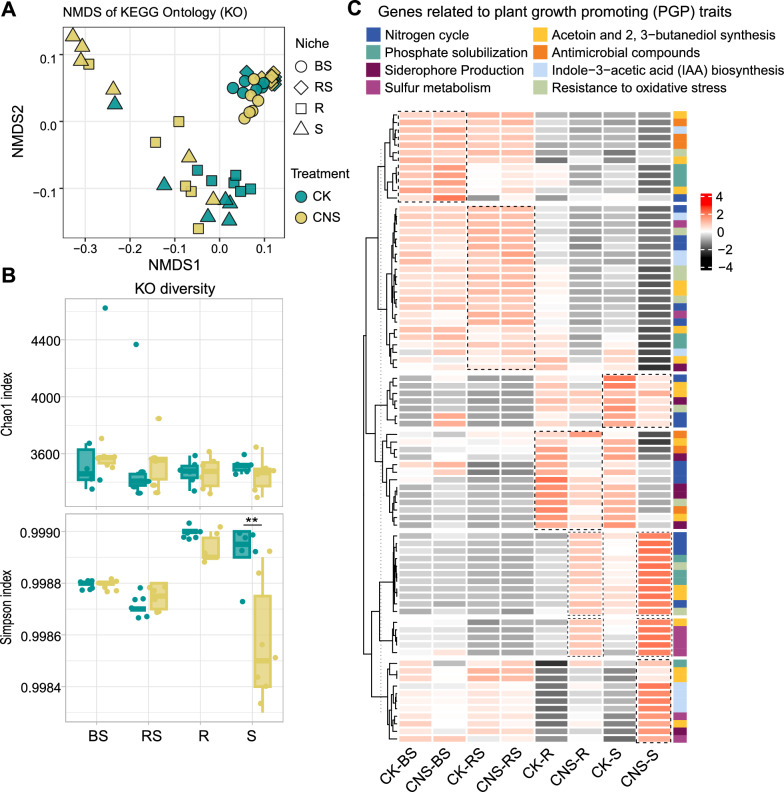
Functional profiles of tobacco microbiomes. **A** Nonmetric multidimensional scaling (NMDS) ordination analysis based on Bray–Curtis distance matrices of the KEGG ontology annotations, showing that the CNS-induced tobacco microbiome significantly differed from the control microbiome (*n* = 48). **B** Boxplot of the functional diversity of microbiomes in four compartments. Asterisks above the boxes indicate a significant difference (*P* < 0.01). **C** Heatmap exhibiting the relative abundance of functional genes associated with plant growth–promotion functions. All genes associated with plant growth–promotion functions are listed in Additional file 2: Table S5. The four compartments are bulk soils (BS), rhizosphere (RS), root endosphere (R), and stem endosphere (S)

To investigate the effect of CNS on the functional diversity of different compartments, we used PICRUSt2 to predict the bacterial metagenome and annotated it with the Kyoto Encyclopedia of Genes and Genomes (KEGG) database. A total of 3,685 KEGG orthologs were predicted in the four tobacco-associated communities. Our NMDS ordination analysis revealed significant differences in the functional profiles between the soil and endophytic communities (Fig. 5A), and CNS also had a significant effect on the functional profiles of the endophytic microbial communities (*P* < 0.01) (Fig. 5A). Based on the KEGG annotations, alpha-diversity analysis showed no significant differences among the functions present in the different ecological niches, except for a significant decrease in the diversity of the functions expressed in the stem endophytes after CNS treatment (Fig. 5B).

Next, we tried to identify genes that might have plant growth-promoting functions based on their KEGG annotation (Additional file 2: Table S5). In the stem endophytic community, the abundance of genes involved in hydrogen sulfide production and sulfate biosynthesis was enriched after CNS treatment, as were functional genes involved in phosphate transport, such as *pstA*, *pstB*, and *pstC*. The genes encoding enzymes involved in nitrogen metabolism, such as glutamate synthase and ferredoxin-nitrite reductase, were found to be more abundant in the root and stem endophytic communities; however, the abundance of genes involved in iron carrier production, such as *enterobactin synthetase* and *diaminobutyrate-2-oxoglutarate transaminase*, decreased after CNS treatment. Some genes involved in the biosynthesis of tryptophan (indole-3-acetic acid (IAA) precursors), such as *anthranilate synthase component II*, *anthranilate phosphoribosyltransferase*, *phosphoribosylanthranilate isomerase*, and *indole-3-glycerol phosphate synthase*, were also observed to be enriched in the stem endosphere. The volatile compounds acetoin and 2,3-butanediol directly affect plant growth by promoting root formation [16], and we found that the genes associated with acetoin production, such as *pyruvate dehydrogenase* (*pdhB*) and *acetolactate synthase* (*ilvH*), were enriched after CNS treatment. The gene encoding *S-(hydroxymethyl) glutathione dehydrogenase* (*adhC*), which was responsible for converting ethylene to 2,3-butanediol, was also enriched after CNS treatment. These analyses indicated that the microbial communities assembled in the tobacco plant endosphere treated with CNS might be involved in enhancing the plant growth-promoting process.

### Plant-beneficial effects of the CNS-enriched microbes

**Fig. 6:**
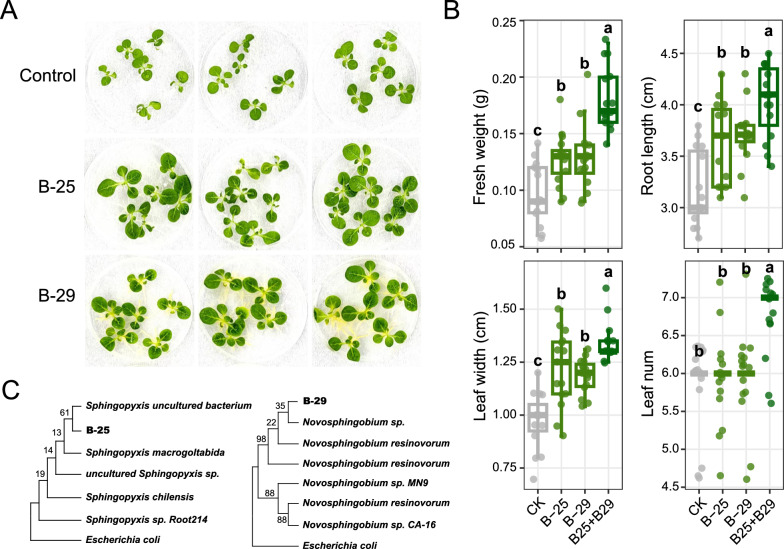
The effect of two isolated bacteria on tobacco growth. **A** Phenotype of tobacco plants after inoculation with two bacteria on MS plates. **B** Effects of single inoculation with B-25 (*Sphingopyxis* sp*.*) or B-29 (*Novosphingobium* sp.) and coinoculation of two strains (B25 + B29) on different tobacco growth parameters. ANOVA with an LSD test (*p* < 0.05) indicated statistically significant differences denoted by different letters for each assessed parameter. **C** Phylogenetic analysis of isolated bacteria. Neighbor-joining trees were constructed using partial 16S rRNA sequences of the two strains and their close relatives. *Escherichia coli* was used as an outgroup for rooting the tree

Finally, we isolated candidate bacteria from the rhizosphere of tobacco after CNS treatment and identified them based on their 16S rRNA sequences. To further characterize whether these isolates could promote plant growth, we planted tobacco on plates preinoculated with the isolated strains. Two of the bacterial strains, B-25 and B-29, significantly promoted tobacco growth (Fig. 6A), increasing plant fresh weight by 31.25% and 24.75%, respectively. In addition, they significantly promoted the aboveground growth and root elongation of tobacco (Fig. 6B). When grown on plates inoculated with B-25, the maximum leaf width of tobacco increased by 26.30% ± 17.6%, and the root length increased by 11.34% ± 12.19%. B-29 also significantly increased the leaf width and plant height of tobacco seedlings. However, there was no significant change in plant height or lateral root density of tobacco under CNS treatment (Additional file 1: Fig. S5A). When both bacterial strains were coinoculated, a remarkable 86.90% ± 27.36% increase in fresh weight was observed (Additional file 1: Fig. S5B). Compared with individual strains, the higher growth-promoting efficacy of coinoculation demonstrated the presence of synergistic interactions among diverse endophytic bacteria (Fig. 6B).

Using the 16S rRNA sequence, our phylogenetic analysis showed that strain B-29 belonged to the genus *Novosphingobium*, with *Novosphingobium resinovorum* being the nearest phylogenetic relative (Fig. 6C). Strain B-25 belonged to *Sphingopyxis* and was most closely related to *Sphingopyxis macrogoltabida*. Notably, these two genera were also significantly enriched in the roots following CNS treatment. These results suggested that CNS might recruit some beneficial microorganisms, thereby enhancing tobacco growth.

To gain insights into the mechanisms of these two bacterial strains, we investigated the presence of genes related to free nitrogen fixation (*nifH*), ACC deaminase production (*acdS*), IAA production (*ppdC*), and DAPG production (*phlD*) in the genomes of B25 and B29. Our results confirmed the presence of IAA biosynthesis genes in the genome of B25 (Additional file 1: Fig. S6).

## Discussion

Trace amounts of CNS have previously been shown to promote nutrient absorption and growth of tobacco under various conditions, such as BY-2 suspended cells [19] and hydroponics [23], demonstrating its potential applications in agriculture. In the present study, we attempted to explore the relationship between CNS and plants from the perspective of nanomaterial–microbe–plant interactions. The application of CNS in the soil promoted tobacco growth and significantly changed the structure and diversity of the root and stem endophytic microbial communities, even increasing the stability of the microbial ecological network to some extent. CNS also increased the abundance of several microorganisms and some functional genes, which might promote plant growth. These results suggest that CNS might benefit plant growth by restructuring the microbial community and specifically enriching beneficial microbes with plant growth–promoting capacity. A better understanding of the relationship between CNS and the recruited beneficial microorganisms could provide new possibilities for the application of carbon nanomaterials and the better utilization of the microbiome to develop more sustainable agricultural practices.

The microbiome is involved in many aspects of promoting plant growth, including increasing nutrient availability and absorption, producing plant hormones and growth-stimulating substances, competing with pathogens for resources, and producing compounds that inhibit pathogens [24]. Generally, some strains of microorganisms, especially those belonging to the genera *Pseudomonas*, *Bacillus*, *Streptomyces* [25], and *Trichoderma* [26], have the ability to promote plant growth and inhibit pathogens in many plant species. *Sphingopyxis* and *Novosphingobium* are considered important genera in various biodegradation and bioremediation applications [27, 28], and they also include a few plant growth-promoting rhizobacteria (PGPR) species [29]. A genomic analysis of *Novosphingobium* bacteria showed that they contained genes related to IAA, acetoin, and siderophore biosynthesis and had the capacity to use a wide range of plant-derived organic compounds [30, 31]. We found that the abundance of *Sphingopyxis* and *Novosphingobium* was higher in the rhizosphere of CNS-treated plants and demonstrated the ability of the isolated strains to promote plant growth. Commonly, plant growth enhancement is a conserved feature among different plant species. The B-29 strain shared high sequence similarity with *Novosphingobium pokkalii*, which was previously described as a plant growth promoter [30, 32, 33]. The B-25 strain belonged to *Sphingopyxis*, which was also previously described as a plant growth promoter [34]. Similarly, biochar treatment led to changes in the composition of the tomato rhizosphere microbiome, including the recruitment of PGPR microorganisms such as *Sphingopyxis* [35]. However, plant growth enhancement is sometimes not a conserved feature among all plant species, and some PGPR strains isolated from one plant even inhibit the growth of another plant [36]. The reason might be that environmental factors and host plants also affect the PGP effect due to the complexity of plant-bacteria symbiosis. Further research is needed for successful cross-species applications of PGPR.

In addition, compared with the control, the abundance of genes in several metabolic pathways with the potential to affect plant growth was significantly altered in the endophytic microbiome after CNS treatment. Mineral nutrient metabolism and acquisition are crucial for plant development, with limited soluble phosphate and nitrogen availability limiting plant growth and survival [37–39]. We detected increased expression of genes associated with nitrogen metabolism and the phosphorus and sulfur cycling pathways in the microbiome after CNS treatment. Compounds produced by microorganisms, such as the plant hormone IAA [40] and VOCs such as acetone and 2,3-butanediol [16], can promote plant growth by stimulating root branching and elongation. The genes involved in the biosynthesis of these compounds were also enriched after CNS treatment. The promotion of plant growth, together with the differences in community composition, therefore indicates that the microbiome may play an intermediary role between the CNS and plant performance. The overall changes in the composition of the endophytic microbiome and the changes in the abundance of specific PGPRs might jointly explain the observed improvement in plant performance in the presence of CNS. Metagenomic and metatranscriptomic analyses are needed to further explore the effect of the CNS on soil microbial function.

Different carbon-based nanomaterials exert different effects on soil bacterial abundance, diversity, and microbial community composition, likely due to their distinct physical structures and surface chemistry or differences in the dose, application time, area, size, and reactivity of the carbon nanomaterials [17, 41]. Research into the effects of carbon nanomaterials on the soil microbiome has yielded conflicting results [42]; several studies demonstrated that carbon nanomaterials might exhibit environmental toxicity, reducing microbial diversity and activity [43], whereas other studies reported either an insignificant effect [44, 45] or even a tendency toward a positive effect on microbial communities [18, 46]. Due to the complexity of interactions between microorganisms and plants, as well as the connection between nanomaterials and plant physiological processes, it is often difficult to explore the mechanism by which the nanomaterials influenced these microbes. The experiments in this study were all conducted in laboratory environments in plant growth chambers, which aimed to simulate natural environments but were unlikely to fully represent the complexity of natural systems. Further field experiments will ultimately be needed to explore the effects of CNS in practical applications, as well as the long-term effect of carbon nanomaterials on soil ecological systems.

## Conclusion

In this study, the effect of CNS on plant growth and the microbiome in soil and endophytes was investigated under laboratory conditions. CNS enhanced tobacco growth and led to shifts in the diversity and composition of the microbial community. Moreover, in the endophytic microbiome, the abundance of some microbial communities and functional genes related to plant growth-promoting performance were increased under CNS treatment. Two bacteria (*Sphingopyxis* and *Novosphingobium*) were isolated, and their effect on promoting the growth of tobacco plants was confirmed. Overall, this study highlighted that CNS had a substantial influence on soil and endophytic microbial communities, and emphasized the intricate relationship between the microenvironment and plant growth. This study enhanced our understanding of the microecological advantages of CNS application within the tobacco ecosystem.

## Materials and methods

### Experimental design

Commercially produced CNS was purchased from Beijing Naisis New Material Technology (Beijing, China) and was prepared using a pulse electrodeposition method using graphite. The particle size ranged from 10 to 100 nm in diameter, with an average size of 30 nm, and the purity was more than 99.9%. The method of CNS preparation was described by Chen et al. [47]. Briefly, two high-purity graphite plates with a purity of 99.999% were immersed in deionized water containing 0.1% ethylene glycol, and voltage was applied to the edge of the graphite plate. The voltage was set to 16 V, and the current was 0.2 A. The solution was continuously stirred on the graphite electrode for several days until the solution turned black at room temperature.

Tobacco (*Nicotiana tabacum L*.) seeds (cv. K326) were surface-sterilized with 10% sodium hypochlorite for 10 min and washed three times with sterile water. The sterilized seeds were germinated on 1/2 Murashige and Skoog (MS) agar plates. After germination, individual 21-day-old tobacco seedlings were transplanted into pots (0.5 L; diameter = 10 cm) containing potting soil mix (horticultural grade peat:vermiculite in a 9:1 vol:vol mixture). One time every three days for nine days, 100 mL of CNS at a concentration of 15 µg/mL was added to each pot, while the control group received distilled water. Each treatment included 15 plant replicates. The plants were grown under normal conditions in a growth chamber (16 h light/8 h dark, 28/25 °C), and the plant growth indicators were measured 15 days after the final treatment.

### Sample collection

We focused on four compartments (BS: bulk soil, RS: rhizosphere soil, R: root endosphere and S: stem endosphere). Bulk soil refers to the soil that was more than 1 mm from the plant roots. The root system and rhizosphere soil (within 1 mm of the root system) were harvested after shaking off the loosely attached soil on the roots. The roots or stems were placed in a 50-mL tube containing 15 mL sterile phosphate-buffered saline (PBS) solution. After sonication at 40 kHz for 1 min, the roots were transferred to a new clean tube. Soil and plant samples were stored in liquid nitrogen and transported to the laboratory. All samples were stored at − 80 °C for DNA extraction.

### DNA extraction and amplicon sequencing

Each sample was divided into four ecological niches: bulk soil (BS), rhizosphere soil (RS), root endosphere (R), and stem endosphere (S). Each treatment group contained six biological replicates, for a total of 48 samples. For the root and stem endosphere samples, 5 g of roots or 10 g of stems were sonicated in 75% ethanol for 5 min, immersed in 1% sodium hypochlorite solution for 5 min, immersed in 75% ethanol for 30 s, and finally washed three times with sterile water to sterilize the surface of the roots and stems. The sterilized roots or stems were ground using sterile mortars and pestles with liquid nitrogen. DNA was extracted from 0.5 g of bulk soil, rhizosphere soil, or ground endosphere samples using the Mag-Bind Soil DNA Kit (Omega Biotek, Doraville, GA, USA) according to the manufacturer’s instructions. The DNA concentration was evaluated using a NanoDrop 1000 spectrophotometer (Thermo Fisher Scientific, Waltham, MA, USA), and the DNA quality was evaluated using gel electrophoresis with a 1.0% agarose gel.

The bacteria were amplified using primers 515F and 806R, targeting the V4 region of the 16S rRNA gene, whereas fungi were amplified using primers ITS1F and ITS2, targeting the ITS1 region. The PCR system and amplification conditions are shown in Additional file 2: Table S1. After purification, the PCR products were used for library construction, and high-throughput sequencing was performed using the MiSeq platform (Illumina, San Diego, CA, USA).

### Amplicon sequencing data analysis

The amplicon sequencing data were processed using QIIME 2 (v2021.11.0) [48]. The DATA2 module was used for quality control, denoising, and chimera filtering. Unique amplicon sequence variants (ASVs) were obtained by clustering the effective sequences with a 97% similarity threshold. The ASVs were then compared to the SILVA (v138) [49] prokaryotic database and the UNITE (v2021.5.10) [50] eukaryotic database for taxonomic annotation. Based on the ASV results from QIIME2 after rarefaction, the relative abundance data were used as the microbial abundance data. The alpha-diversity indices, including the Shannon and Chao1 indices, were estimated with the ASV table in QIIME2 and then visualized in box plots. The Beta-diversity of the bacterial communities was evaluated by calculating the Bray–Curtis distance matrix and was visualized using principal coordinate analysis plots. The potential functional profiles of the bacterial communities were predicted using PICRUSt2 (Phylogenetic Investigation of Communities by Reconstruction of Unobserved States) based on the 16S rRNA gene data [51].

### Co-occurrence network analysis

To determine the co-occurrence network of the microbial communities and the structural differences of the microbial communities in each ecological niche, a Spearman correlation analysis was performed between the ASVs (with a sample size greater than half of the total samples and an abundance greater than 0.001), and data with a correlation coefficient greater than 0.6 and *P* < 0.05 were visualized using Gephi (v0.9.7) software [52]. Nodes represent individual ASVs, and edges represent the correlations between nodes in the microbial community network.

### Statistical analysis

All statistical analyses were performed using R (v4.2.0) (The R Foundation for Statistical Computing, Vienna, Austria). Alpha-diversity indices (Shannon index, Chao1 index, and Simpson index) of the bacterial community were calculated in QIIME 2. Beta-diversity of both bacterial and fungal communities was assessed by computing weighted UniFrac distance matrices and then ordinated using nonmetric multidimensional scaling (NMDS). The PERMANOVA test was performed using the Adonis function in the “vegan” package to evaluate the relative contribution of different factors to community differences. Differential abundance analysis between microbiomes was calculated using the DESeq2 R package [53]. LefSe analysis between each pair of CNS-applied and control samples were performed at the genus level. Under one-against-all comparison mode, each genus with an α less than 0.05 and an LDA score greater than 2 was defined to be significantly different between rhizosphere and soil. A nonparametric Kruskal‒Wallis test was performed to evaluate the alpha-diversity difference and the taxonomical difference among different treatments. One-way analysis of variance (ANOVA) followed by the LSD test was used to determine the statistical significance of the growth index among different groups.

### Isolation and identification of tobacco rhizosphere bacteria

Bacteria were isolated from the rhizosphere of tobacco plants treated with CNS using tryptic soy broth (TSB) solid medium and the plate dilution culture method. The isolated bacteria were purified by streaking on solid medium. The V5–V7 region of 16S rRNA was amplified using primers 799F and 1193R, and the sequences were identified by comparing them to the SILVA database after Sanger sequencing. The strains were stored in glycerol at − 80 °C. A multiple sequence alignment was performed using MEGA X software [54], and the phylogenetic tree was constructed using the neighbor-joining method. The bootstrap resampling method with 1,000 iterations was employed to assess the statistical significance of the phylogenetic tree.

### Plant growth–promotion assay

A single colony was picked and inoculated into 1/2 TSB liquid medium and then shaken at 28 °C and 160 rpm for 24–48 h. The logarithmic phase bacterial suspension was collected, and the concentration was adjusted to an optical density (OD)600 value of 0.5. The B-25 strain was added to the B-29 strain in equal proportions as a coincubation. Five-milliliter suspensions of each kind of strain were mixed for coincubation. A 50-µL aliquot of the bacterial solution was spread on a 90-mm 1/2 MS plate. Three replicates were performed for each strain, and sterile TSB was used as a control. Tobacco seedlings were surface-sterilized and grown on MS medium for 20 days. They were then transferred to the bacterium-inoculated medium, with five seedlings per plate. The plates were randomly placed in a growth chamber and grown for 10 days at 21 °C under 16-h light/8-h dark conditions. Plant morphological indicators, such as fresh weight, plant height, lateral root density, root length, number of leaves, and maximum leaf width, were measured.

### Molecular detection of plant-beneficial functional genes

PCRs and 1% agarose gel electrophoresis were conducted to detect the presence of genes related to plant growth-promoting function in the genomes of the two bacterial strains B-25 and B-29. Four category genes were analyzed: *nifH* (involved in free nitrogen fixation) [55], *acdS* (cleaves of the ethylene precursor 1-aminocyclopropane-1-carboxylate (ACC)) [56], *phlD* (polyketide synthase gene, produces the auxinic-type root-branching signal 2,4-diacetylphloroglucinol (DAPG)) [57] and *ppdC* (phenylpyruvate decarboxylase gene, involving 3-indole acetic acid (IAA) production)[58]. Primer sequences can be found in Additional file 2: Table S1.

### Supplementary Information

**Additional file 1: Figure S1.** NMDS ordinations based on weighted UniFrac distance matrices of bacterial and fungal communities (n=48). **Figure S2.** Venn diagram depicting number of enriched or depleted ASVs in each compartment. **Figure S3.** Differential abundance between CNS-treated and control sample. **Figure S4.** Bacterial-fungal interkingdom networks. **Figure S5.** The effect of two isolated bacteria on tobacco growth. Figure S6. Gel electrophoresis of plant growth-promoting corresponding gene fragments.

**Additional file 2: Table S1.** Primer sequences and PCR amplification conditions in this study. **Table S2. **The mean relative abundance of bacterial and fungal communities at the phylum level in the CNS-treated and control samples of four compartments. **Table S3.** Differentially abundant analysis showing the enriched and depleted genus in the CNS applied plants compared with control. **Table S4.** The hub nodes of microbial interkingdom co-occurrence networks. **Table S5. **Genes related to plant growth promoting (PGP) traits.

## Data Availability

The raw sequence data reported in this paper have been deposited in the Genome Sequence Archive (Chen et al., 2021) in BIG Data Center (Members and Partners, 2022), Beijing Institute of Genomics (BIG), Chinese Academy of Sciences, under accession numbers CRA017876, which can be publicly accessible at http://bigd.big.ac.cn/gsa.
