# Supplementary material for: Carbon nanosol-induced assemblage of a plant-beneficial microbiome consortium
Source: J Nanobiotechnology. 2023 Nov 20;21:436. doi: 10.1186/s12951-023-02213-6 (PMC10658824; doi:10.1186/s12951-023-02213-6)
Supplement: Supplementary file 1 — Additional file 1: Figure S1. NMDS ordinations based on weighted UniFrac distance matrices of bacterial and fungal communities (n=48). Figure S2. Venn diagram depicting number of enriched or depleted ASVs in each compartment. Figure S3. Differential abundance between CNS-treated and control sample. Figure S4. Bacterial-fungal interkingdom networks. Figure S5. The effect of two isolated bacteria on tobacco growth. Figure S6. Gel electrophoresis of plant growth-promoting corresponding gene fragments. [file 12951_2023_2213_MOESM1_ESM.docx]

Lingtong Cheng^1,2^, Jiemeng Tao^1,2^, Zechao Qu^2^, Peng Lu^1,2^, Taibo Liang^3^, Lijun Meng^2^, Wei Zhang^4^, Nan Liu^4^, Jianfeng Zhang^1,2^, Peijian Cao^1,2,5,*^, Jingjing Jin^1,2,*^

^1^Beijing Life Science Academy, Beijing 102200, China

^2^China Tobacco Gene Research Center, Zhengzhou Tobacco Research Institute of CNTC, Zhengzhou 450001, China

^3^Key Laboratory of Ecological Environment and Tobacco Quality, Zhengzhou Tobacco Research Institute of CNTC, Zhengzhou 450001, China

^4^China National Tobacco Quality Supervision & Test Center, Zhengzhou, 450003, China

^5^School of Agricultural Sciences, Zhengzhou University, Zhengzhou 450001, China

**
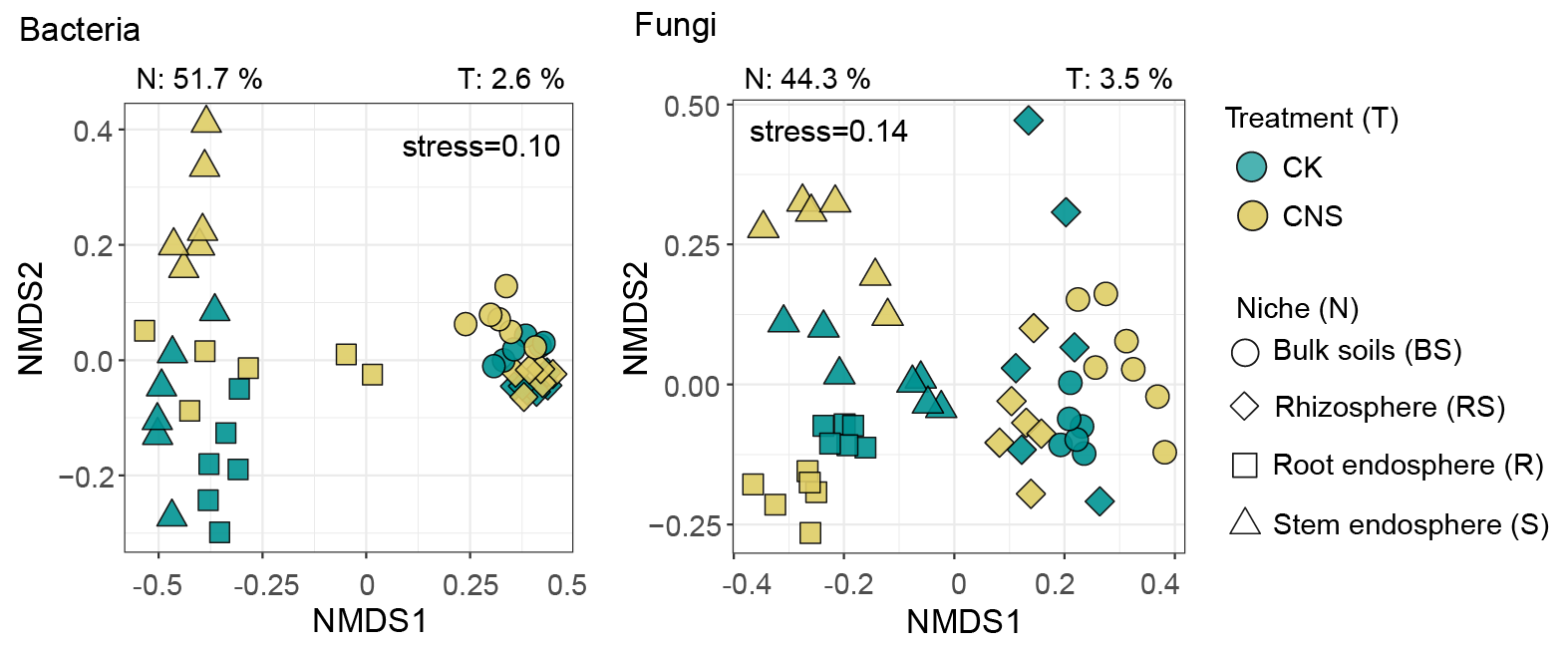
**

**
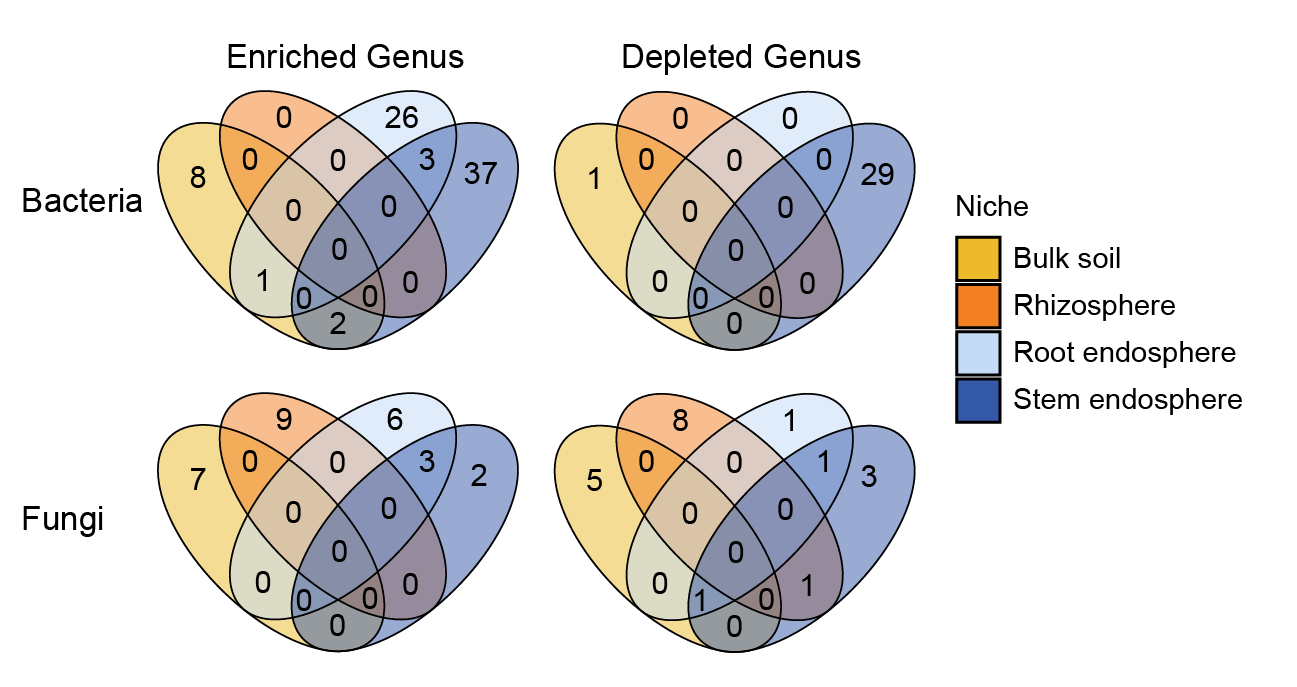
Figure S1. NMDS ordinations based on weighted UniFrac distance matrices of bacterial and fungal communities (n=48)**

**Figure S2. Venn diagram depicting number of enriched or depleted ASVs in each compartment**

**
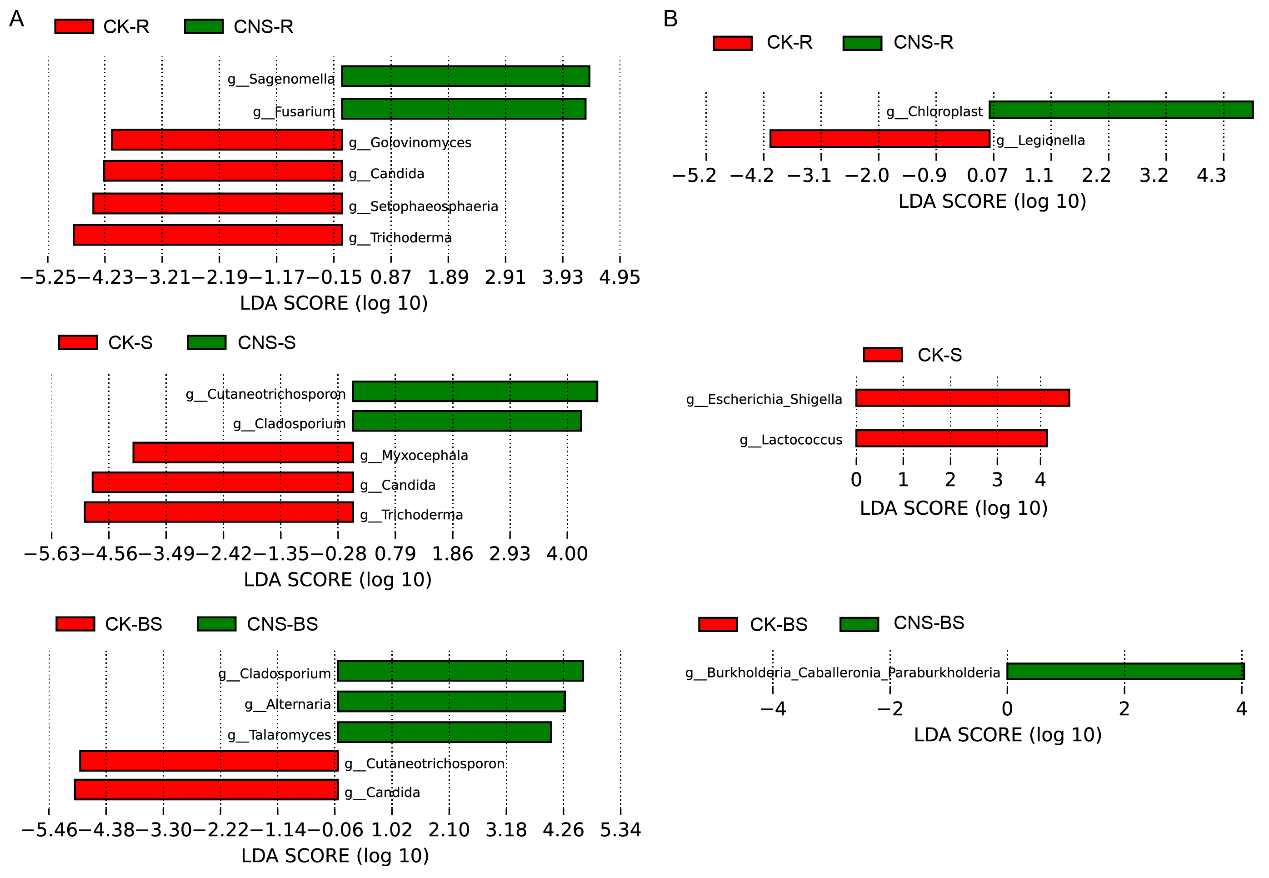
**

**Figure S3. Differential abundance between CNS-treated and control sample.**

(A) The LEfSe analysis results of fungi at genus level by linear discriminant analysis (LDA) > 4. A negative LDA score represents depletion in CNS and enrichment in control (red) and a positive LDA score represents the opposite (green).

(B) The LEfSe analysis results of bacteria at genus level by linear discriminant analysis (LDA) > 4.

**
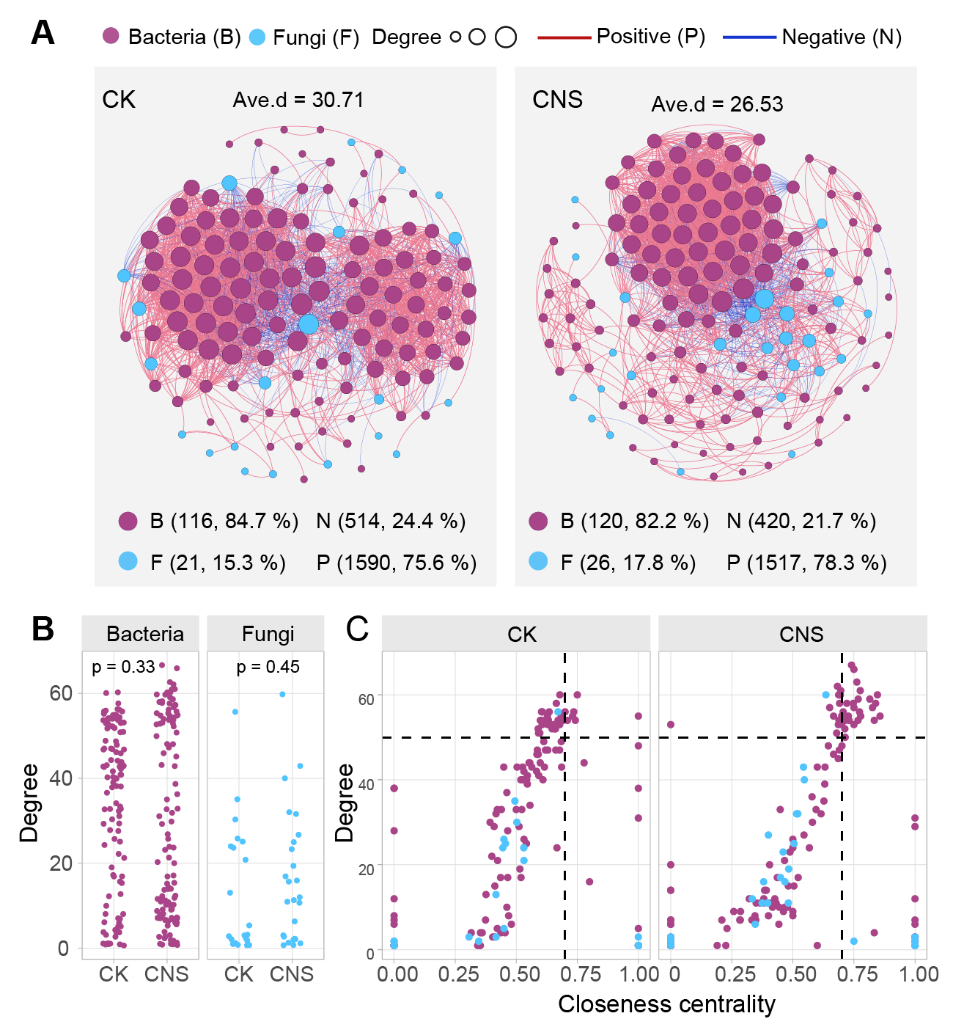
**

**Figure S4. Bacterial-fungal interkingdom networks.**

(A) Co-occurrence network analysis of full dataset (n=48) showing microbial interkingdom network patterns affected by CNS.

(B) Comparison of degree for bacterial and fungal taxa between control and CNS treated samples.

(C) Comparison of closeness centrality between control and CNS treated samples.


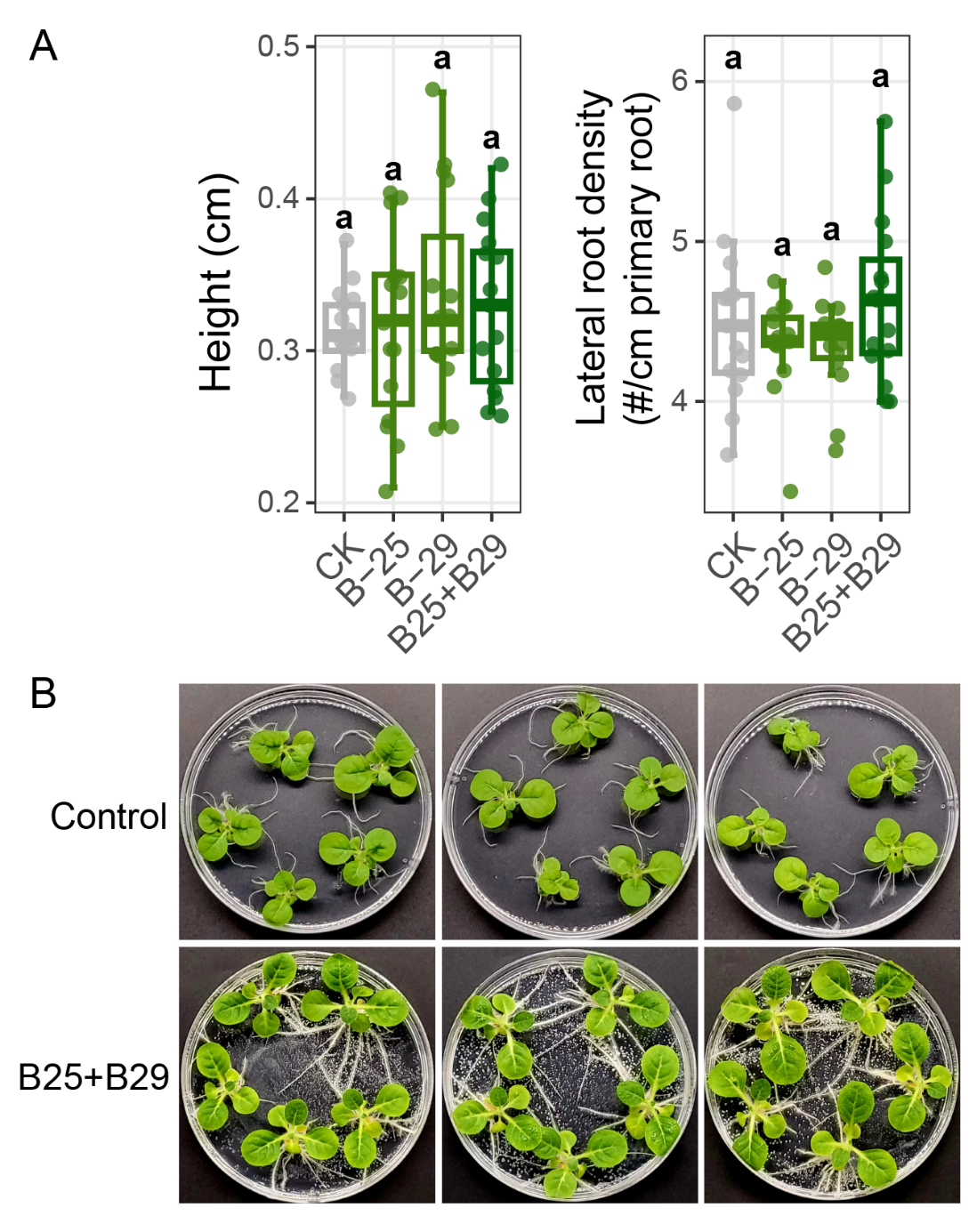
**Figure S5. The effect of two isolated bacteria on tobacco growth.**

(A) Effects of single-inoculation with B-25 (*Sphingopyxis sp.*) or B-29 (*Novosphingobium sp.*) and coinoculation of these two strains (B25+B29) on height and lateral root density. ANOVA with an LSD test (*p* < 0.05) indicated statistically significant differences denoted by different letters for each assessed parameter.

(B) Phenotype of tobacco plants after coinoculation with two bacteria on MS plates.


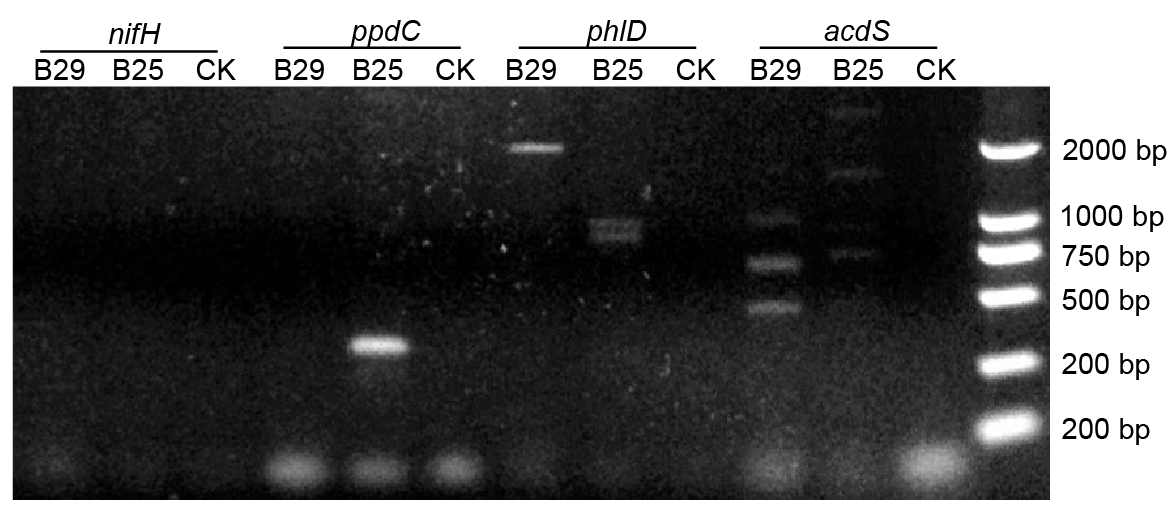
**Figure S6. Gel electrophoresis of plant growth-promoting corresponding gene fragments.**

The fragments of four genes (*nifH*, *acdS*, *ppdC*, *phlD*) amplified from DNA of strains B-25 and B-29 using specific primers were subsequently detected via gel electrophoresis. DL2000 was used as the molecular weight marker.
